# Rose Bengal Induced Photothrombosis in CAM Integrated Human Split Skin Grafts—A Feasibility Study

**DOI:** 10.3390/ijms24043689

**Published:** 2023-02-12

**Authors:** Georg Kornhäusel, Christian Smolle, Kathrin Kreuzer, Lars-Peter Kamolz, Nassim Ghaffari-Tabrizi-Wizsy

**Affiliations:** 1 University Clinic of Paediatric and Adolescent Surgery, Medical University of Graz, 8036 Graz, Austria; 2 University Clinic of Surgery, Division of Plastic, Aesthetic and Reconstructive Surgery, Medical University of Graz, 8036 Graz, Austria; 3 Otto Loewi Research Center, Department of Immunology, CAM Lab, Medical University of Graz, 8036 Graz, Austria; 4 COREMED—Center for Regenerative Medicine, Joanneum Research Graz, 8010 Graz, Austria

**Keywords:** Rose Bengal, wound healing, wound healing models, chick chorioallantoic membrane

## Abstract

Wound healing is a complex process requiring an adequate supply of the wound area with oxygen and nutrients by neo-vascularization, to renew tissue. Local ischemia can result in the formation of chronic wounds. Since there is a lack of wound healing models for ischemic wounds, we aimed to develop a new one, based on chick chorioallantoic membrane (CAM) integrated split skin grafts and induction of ischemia with photo-activating Rose Bengal (RB) in a two-part study: (1) investigation of the thrombotic effect of photo-activated RB in CAM vessels and (2) investigation of the influence of photo-activated RB on CAM integrated human split skin xenografts. In both study phases, we observed a typical pattern of vessel changes after RB activation with a 120 W 525/50 nm green cold light lamp in the region of interest: intravascular haemostasis and a decrease in vessel diameter within 10 min of treatment. In total, the diameter of 24 blood vessels was measured before and after 10 min of illumination. Mean relative reduction of vessel diameter after treatment was 34.8% (12.3%–71.4%; *p* < 0.001). The results indicate that the present CAM wound healing model can reproduce chronic wounds without inflammation due to the statistically significant reduction of blood flow in the selected area using RB. Combined with xenografted human split skin grafts, we established the set up for a new chronic wound healing model for the research of regenerative processes following ischemic damage of the tissue.

## 1. Introduction

Since the prevalence and incidence of chronic wounds are increasing [1], it is important to study the role of ischemia in human wound healing. Inflammation and ischemia play the main role during regenerative processes. At the moment, in-vivo models in research may be the best options available with regard to the similarity in the whole regenerative process. However, it is not yet possible to only study the influence of ischemia. This is because the inflammation process starts immediately after injury and dominates the affected area. Being superior to classic in-vitro models, the chick chorioallantoic membrane not only represents an alternative to animal testing but also does not raise ethical or legal questions or violate animal protection laws [2]. Furthermore, because of the lack of innervation during the respective timeframe of embryonal development, the embryos experience no pain [3]. Due to the primarily lack of the chick embryos’ immune system,T- and B-lymphocytes start to appear at day 11 of embryonic development [3], and the innate immune system does not become mature until day 21. The chick embryo serves as a naturally immunodeficient host capable of sustaining grafted tissues and cells without species—specific restrictions [4]. Rose Bengal (RB)—4,5,6,7-tetrachloro-2’,4’,5’,7’-tetraiodofluorescein—is a halogenated xanthene dye. It is mostly used as a photosensitizer in biological and non-biological systems [5]. After activation through green light illumination, RB generates radical oxygen species (ROS) [6], which destroy the endothelial cells and activate the clotting system to produce thrombosis. RB has therefore been used in many mouse and rat studies to induce photothrombotic cortical lesions [7,8,9,10]. Considering the fact that the supply of the wound area with oxygen and nutrients through local vasculature are the crucial steps for adequate wound healing, this study aims to review methods for the implementation of a new model that allows inducing localized ischemia with a 120 W 525/50 green cold light lamp in selected areas of the CAM with and without human split-thickness skin grafts.

## 2. Results

### 2.1. Effects of RB in 5-Day-Old Chick Embryos

**Figure 1:**
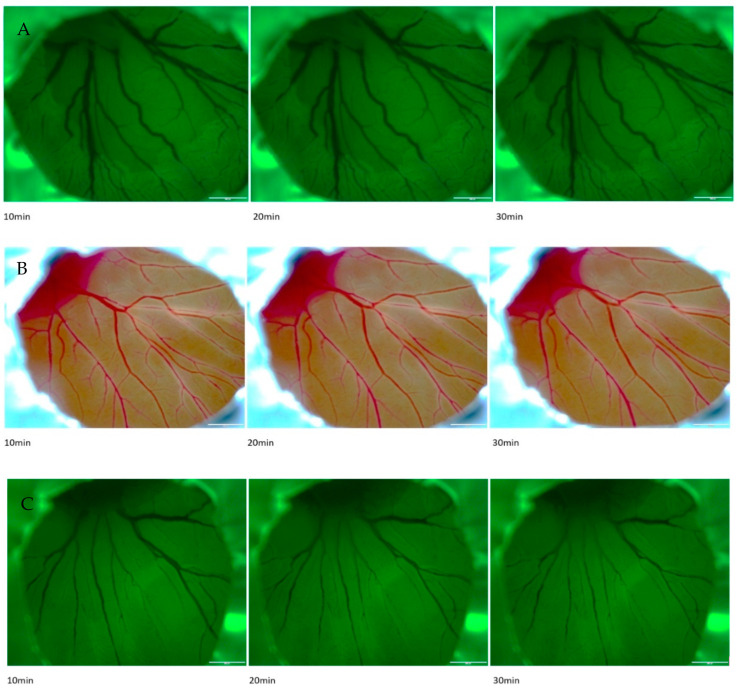
Effects of Rose Bengal in 5-day-old chicken embryos. Representative images of (**A**) injection of 3 µL of PBS (negative control) and illumination for 30 min with 120 W 525/50 green cold light, (**B**) injection of 3 µL of a solution of Rose Bengal of 50 mg/mL concentration without illumination for 30 min with 120 W 525/50 green cold light and (**C**) injection of 3 µL Rose Bengal with illumination with 120 W 525/50 green cold light.

The photothrombotic effects of RB were first tested on 5-day-old chick embryos after injection of 3 µL of 50 mg/mL RB in PBS solution and subsequent irradiation with green light (525/50 nm filter; Figure 1C). 

After 10 min of irradiation a statistically significant reduction of vessel diameter could be observed. No significant decrease in vessel diameter was observed in CAMs after RB injection without irradiation (Figure 1B) as well as after injection of 3 µL of PBS (phosphate buffered saline) and irradiation with green light (Figure 1A).

### 2.2. Effects of Rose Bengal with Illumination in CAM

**Figure 2:**
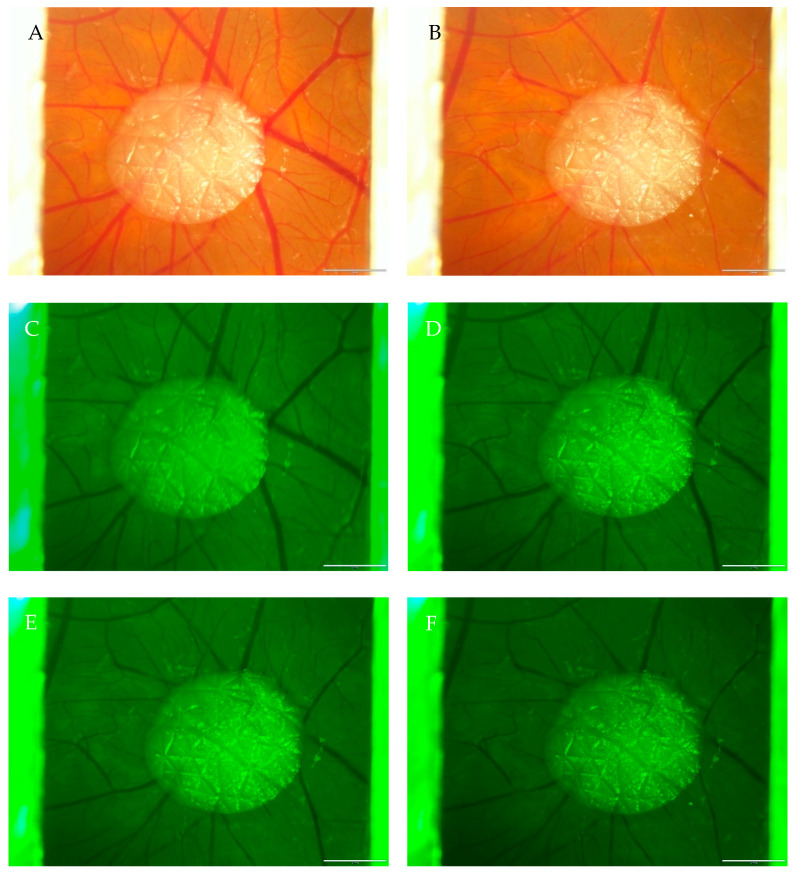
Rose Bengal induced vessel changes in CAM with split skin grafts (**A**) before and (**B**) after 10 min of illumination. (**C**) Illumination process t—0 min, (**D**) illumination process t—4 min, (**E**) illumination process t—8 min, (**F**) illumination process t—10 min.

In all the injection experiments conducted, the same concentration of Rose Bengal was used, and the typical pattern of vessel changes could be observed. The blood vessels show stasis throughout the illumination process and, therefore, minimal dilatation during the first 4 min of illumination with a green light lamp (525/50 nm filter). After 8 min of illumination, an apparent reduction of vessel density and a visible decrease in the vessel diameter (Table 1 and Table 2) could be seen. After 10 min of treatment, no further noticeable changes could be observed. Figure 2A–F shows one out of six replicate series with/without transplanted human split skin grafts. In Figure 2A, the selected area with the skin graft can be seen before the illumination process with green light, and in Figure 2B after. All the vessels around the split skin graft in the illuminated area show an apparent lumen diameter reduction. Figure 2C–F shows vessel changes throughout the illumination process of 10 min.

## 3. Discussion

In the present study, we investigated the effect of Rose Bengal on blood vessels using the chick egg chorioallantoic membrane assay in order to implement a new model for ischemic wounds for wound healing research. The major step for implementing our wound healing model was the injection of Rose Bengal in the blood vessels of the chick embryo. By using the ex-ovo method of chicken embryos [11], we could get better access to the blood vessels and the whole CAM. The ideal injection time is about day 10–11 of embryonal development, since then, the chicken vessels are big enough to be punctuated, whilst the chorioallantoic membrane is thin enough to get through.

Rose Bengal is a photosensitive substance that can easily be activated by using a laser or cold light lamp. When activated, it produces ROS, which destroy endothelial cells of the vessels and therefore induces the blood clotting cascade, forming thrombosis [6]. Because of this phenomenon, we decided to use Rose Bengal since it enables the induction of local thrombosis, subsequently ischemia, without any effects on the rest of the organism. Only used in mouse and rat models for studying ischemic stroke, we were the first to use Rose Bengal in CAM. Since there are significant differences between mouse and rat models and the CAM, in particular a matured organism versus a developing one, we not only had to try different dosages and the induction of thrombosis, but also had to find out if Rose Bengal shows safety and tolerability in the developing chicken embryo. In “In vitro study for staining and toxicity of rose bengal on cultured bovine corneal endothelial cells” by Lee Y.C. et al., published 1996, an intrinsic toxicity of Rose Bengal on endothelial cells, e.g., cell swelling, intracytoplasmic vacuole formation, cell detachment, and lysis, augmented by light illumination was described [12]. In our model, we injected 3 µL (150 µg) of a Rose Bengal solution of 50 mg/mL concentration about the concentrations used in mouse and rat models. With considerations about a possible dosage-dependent toxicity we used less. However, half of our treated chicken embryos survived 9 h; other half died about 2 h after the injection process. This was the reason we assume certain toxicity or intolerance of Rose Bengal, maybe dosage dependent, in chicken embryos.

After the injection process, bleeding from the punctured vessel appeared. At this stage of chicken development, only a few millilitres of blood loss lead to death. By means of testing different blood-stopping substances, we finally chose little sponges with m-doc—oxidised cellulose—that immediately stops superficial bleeding without any influence on the organism (see Supplementary Figure S2). The sponges not only stop bleeding but absorb the blood and can be easily removed afterwards, even a few days after. Since this method has not been used before, we cut CAM blood vessels of some chicken embryos with a scissor—to produce huge bleeding—and treated the bleeding with little sponges, including m-doc. In about 2 s, the bleeding stopped, and with the sponge in place we put the chick embryos back into the incubator to observe their survival. Many of the treated chick embryos survived till day 14 of incubation without any problems.

Concerning “Photothrombotic ischemia: a minimally invasive and reproducible photochemical cortical lesion model for mouse stroke studies” by Vivien Labat-gest et al. [13], a cold light lamp with a 525/50 nm filter was taken for the activation of Rose Bengal. The set up for all photos that were taken with our microscope during the process was adjusted at the very beginning of each experimental series—before illumination was started. The chosen CAM area was illuminated for 12 min. After 10 min, the maximum effect could be observed. In all the treated CAMs, the same pattern of changes appeared: reduction of the diameter of the blood vessels and spastic appearance.

To verify the visible reductions of blood vessels, we measured the diameter of four representative ones before and 10 min after the whole process with the program cellSens from Olympus. As shown in the results, the data selected show a reduction of vessel lumen on average of about 34.84% (12.29–71.37%; Table 1 and Table 2). To rule out that the measured vessel changes appear due to a greater distance of the chosen vessel to the microscope throughout the illumination process, only vessels that are fixed on the surface of the CAM were chosen. In addition to this, it is essential to say that the CAM also sticks to the edges of the weighting boats making movements of surface vessels impossible.

A two-sample t-test for dependent samples (paired comparison test) also showed that the decrease in the blood vessel diameter after the treatment with Rose Bengal is statistically significant (*p*-value 8.27 × 10−9, α-value 0.05) and cannot be the effect of the standard deviation (Table 3). 

## 4. Materials and Methods

The Ethics Committee of the Medical University of Graz approved all protocols regarding experiments with human split skin and the CAM. Informed consent was obtained from all subjects (i.e., the split skin donors). All methods were performed in accordance with the relevant guidelines and regulations.

**Ex-Ovo CAM preparation.** Fertilized white Lohman chicken eggs were obtained from a local hatchery (Schropper GmbH, Gloggnitz, Austria). According to the method established by Deryugina EI et al. [11], ex-ovo chick embryos were prepared on day 3 of embryonal development. With the help of a small angle grinder, a 0.5–1.0 cm cut was made on the underside of the egg to finally crack the eggs and transfer the embryo from its in- to ex-ovo state in prepared weighting boats. The used weighting boats had to be washed or dipped in 75% EtOH and dried under UV light for 15 min in a sterile workbench. Once the chick embryos had been transferred, the weighting boats were covered with a square petri dish. A heartbeat, as well as a primitive vessel system, proved the viability of the embryos. Embryos were incubated at 37.6 °C and 50–70% humidity throughout the experiments.

**Split thickness skin graft transplantation.** In our experiments, we used split skin from a 53-year-old female patient undergoing abdominoplasty at the Division of Plastic, Aesthetic and Reconstructive Surgery, LKH Univ.-Klinikum Graz. A split-thickness skin was harvested from the resected skin portion with an electric dermatome. After removal, the split skin grafts were immediately stored in phosphate-buffered saline at 5 °C. Skin samples were produced using a 5 mm biopsy punch. The samples were directly transferred to the CAM of 7-day-old chicken embryos (see Supplementary Figure S1). Xenografting human split skin grafts on the CAM was based on previous studies by Winter R. et al. [14]. The chick embryos were sacrificed the latest on day 14 by decapitation after anaesthesia by placing them on ice for 5 min.

**Injection of Rose Bengal in CAM vessels.** All preparations were conducted under sterile conditions. For the pre-tests, RB was injected in 5-day-old vessels of the yolk sack (Figure 1A–C). All other experiments have been performed on the CAM of 11-day-old chicks.

1. A solution of Rose Bengal in phosphate-buffered saline with a concentration of 50 mg/mL was prepared.
2. 3 µL of the solution was drawn up a pipette and transferred into an Eppendorf tube.
3. A glass capillary was taken and put with the bottom end in the Eppendorf tube so that the solution moved up the capillary by cohesion and adhesion.
4. The filled glass capillary was the injection system, and the ideal blood vessel with a diameter of about 40–80 µm was chosen.
5. The next steps had to be performed under the microscope. With the help of a tweezer, the vessel was carefully lifted and fixed while the vessel was punctured with the glass capillary.
6. The solution was slowly injected by blowing air into the injection system.
7. To stop bleeding, oxidized cellulose was used.
8. It took about 5 s until the solution of Rose Bengal was distributed in the whole vascular system of the chicken embryo and CAM.
9. A cold light illuminator with a wavelength of about 549 nm—the absorption peak of Rose Bengal—and 120 W power was used for photo-activation.
10. The chosen vessel was then illuminated for 10 to 30 min.

**Haemostasis Method.** To stop bleeding after the injection process, oxidized cellulose, also known as m-doc, was used in the form of little sponges (see Supplementary Figure S2). Taken with a tweezer and pressed onto the bleeding vessel right after the injury, haemostasis can be achieved within seconds. Afterwards, removing the sponge is possible and does not evoke new bleeding.

**Figure 3:**
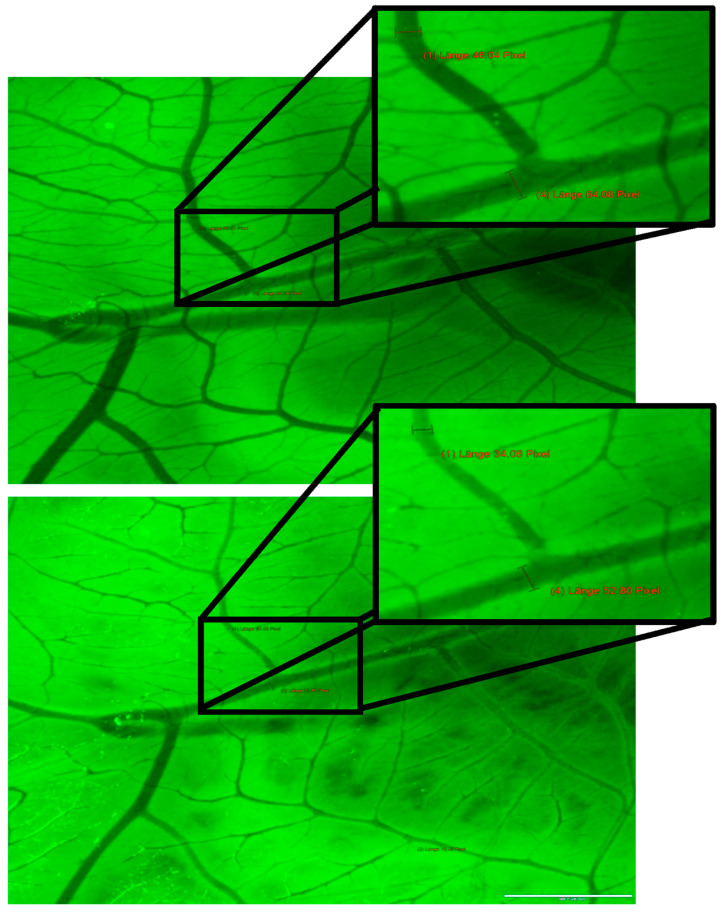
Example of vessel lumen assessment before (**A**) and after 10 min (**B**) of illumination. The white spots mark vessels with apparent changes in vessel diameter.

**Image assessment of Rose Bengal induced vessel changes.** We used the image program “cellSens Dimension 1.12” from Olympus Corporation to assess vessel changes in Rose Bengal treated chick embryos. We analysed the diameter of four representative blood vessels in our recorded pictures of each series before as well as 10 min after illumination and compared the changes (Figure 3). Images that were taken during the illumination process appear in green light. 

**Analysis of Rose Bengal induced vessel changes in CAM.** Four representative vessels in each of the six series were measured, and the results were statistically analysed afterwards. For the statistical analysis, we used the program SPSS and Excel 365 from Microsoft Office (Table 1, Table 2 and Table 3).

## 5. Conclusions

In conclusion, the injection of Rose Bengal in CAM blood vessels, the haemostasis of bleeding, and the induction of photothrombosis are successfully implemented and described in the present study. Only the follow-up of the ischemic area was not yet possible since the chicken embryos only survived hours after treatment, but not longer than one day.

Since all single steps of our experiments—e.g., grafting human skin on CAM, injection of PBS, haemostasis by m-doc, and the illumination process without Rose Bengal—were tolerated by the chick embryos, we assume a certain dosage-dependent toxicity of Rose Bengal. Therefore, toxicity testing of Rose Bengal in chicken embryos has to be completed before further experiments on developing our wound healing model can take place. In addition to this, a dose-finding study could be conducted. Another reason for death after the treatment could be blood loss after an injection. Even though quick haemostasis by m-doc sponges was possible, some millilitres of blood loss could not be prevented.

An essential aspect for the success of our wound healing model is the follow-up of the Rose Bengal treated and illuminated CAM area. Future studies must show whether the treated area becomes necrotic or induces neovascularization. After proving neovascularisation, a new series of experiments, including human split skin transplants, can be undertaken to implement our wound healing model, allow a follow-up until day 14 of incubation, and test the influence of substances on ischemic wounds.

Split skin grafts contain epidermis and dermis, but unlike full-thickness skin, it heals more easily. Re-vascularization occurs faster, making it suitable for successful xenografting onto the CAM, especially for the experimental short timeframe.

Although there are many different models for wound healing in research, every model has its limitations. The only limitations of our wound healing model are the time window of 14 days, the lack of the immune system, the similarities to human beings and the lack of maturity of the organism. Since CAM experiments are only allowed for 14 days of embryological development—afterwards, they are officially seen as animal testing—it is impossible to use our model for long-term studies. Another important aspect is the lack of the immune system. Since the wound healing process consists of many different interacting cells, chemical substances, and unique environmental conditions, our model can only be used to study the effect of therapies or new substances on single aspects of the wound healing process. It is impossible to study the complex interactions of the regeneration process when the immune system is absent.

Furthermore, it is known that the best model for studying human skin regeneration at the moment seems to be the pig model, as it provides the majority of similarities. Nevertheless, it is proved that even animal models do not reflect the wound healing process in the same way as it takes place in the human species. With regards to our model, this means that observed phenomena can differ as well from humans. The last thing to remember is that our model is based on a developing embryological organism. During embryological development, biological processes often differ from those in mature organisms, e.g., because of different expression of genes. Therefore, other reactions of our model concerning testing substances or therapies may occur.

The results of our feasibility study set a foundation for a chronic wound healing experimental setup by specific inducing ischemia. Based on these results, further experiments are possible and needed to study and characterize the effect of Rose Bengal on the complex mechanisms of wound healing.

## Figures and Tables

**Table 1:** Vessel Lumen Assessment.

CAM	Measured Vessel	Diameter in Pixels (0 min)	Diameter in Pixels (10 min)	Ratio (Before/After)
CAM 1	vessel nr. 1	46.04	34.06	1/0.74
	vessel nr. 2	37.01	19.42	1/0.52
	vessel nr. 3	43.00	23.09	1/0.54
	vessel nr. 4	64.08	52.08	1/0.81
CAM 2	vessel nr. 1	19.31	11.18	1/0.58
	vessel nr. 2	34.53	22.20	1/0.64
	vessel nr. 3	20.12	11.18	1/0.56
	vessel nr. 4	41.87	36.24	1/0.87
CAM 3	vessel nr. 1	46.53	30.41	1/0.65
	vessel nr. 2	42.20	12.08	1/0.29
	vessel nr. 3	38.08	13.60	1/0.36
	vessel nr. 4	30.41	18.44	1/0.61
CAM 4	vessel nr. 1	16.12	11.40	1/0.71
	vessel nr. 2	14.56	07.00	1/0.48
	vessel nr. 3	16.00	11.18	1/0.70
	vessel nr. 4	14.14	10.20	1/0.72
CAM 5	vessel nr. 1	56.08	49.19	1/0.88
	vessel nr. 2	37.12	23.35	1/0.63
	vessel nr. 3	18.44	12.00	1/0.65
	vessel nr. 4	37.66	27.17	1/0.72
CAM 6	vessel nr. 1	42.94	33.12	1/0.77
	vessel nr. 2	18.36	13.93	1/0.76
	vessel nr. 3	32.45	25.24	1/0.78
	vessel nr. 4	36.40	24.84	1/0.68

**Table 2:** Overview of Vessel Lumen Assessment.

Maximum of Vessel Lumen Reduction	Minimum of Vessel Lumen Reduction	Arithmetic Mean of Vessel Lumen Reduction	Number of Vessels Measured
71.37%			
	12.29%	34.84%	24
		

**Table 3:** Statistical Analysis of Vessel Lumen Reduction.

	Before Treatment	After Treatment
Mean		
	33.48	22.19
Variance	189.53	148.50
Observations	24	24
Pearson correlation	0.88	
Hypothetical difference of the mean	0	
Degrees of freedom (df)	23	
T-statistics	8.45	
*p*-value (bilaterally)	0.0000000165	
Critical t-value bilaterally	2.07	

## Data Availability

The datasets generated during and/or analysed during the current study are available from the corresponding author upon reasonable request.

## Supplementary Materials

The supporting information can be downloaded at https://www.mdpi.com/article/10.3390/ijms24043689/s1.
