# Supplementary material for: Rose Bengal Induced Photothrombosis in CAM Integrated Human Split Skin Grafts—A Feasibility Study
Source: Int J Mol Sci. 2023 Feb 12;24(4):3689. doi: 10.3390/ijms24043689 (PMC9967479; doi:10.3390/ijms24043689)
Supplement: Supplementary file 1 [file ijms-24-03689-s001.zip › ijms-2057925-supplementary.pdf]

Supplementary Material

Technical Note

Rose Bengal Induced Photothrombosis in CAM Integrated Human Split Skin Grafts—A Feasibility Study

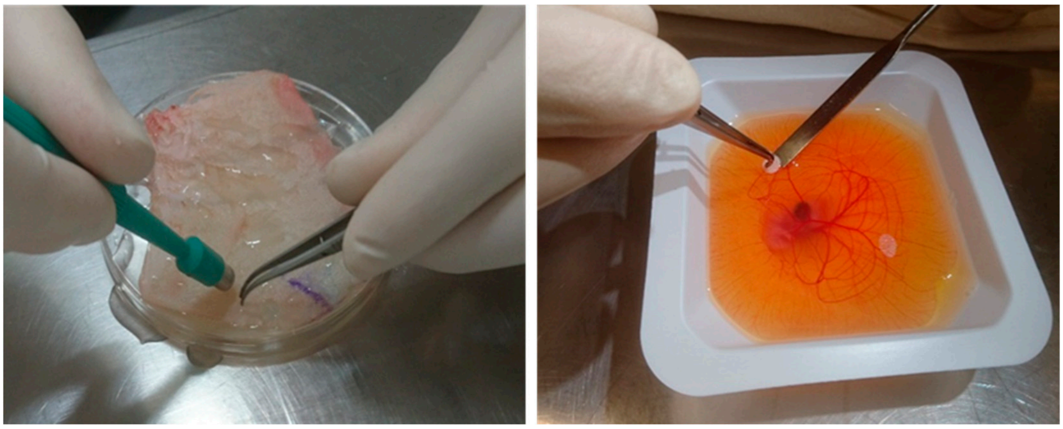

Figure S1. Xenografts of human split skin on the CAM on day 7. In Figure S1 (A)punching of split skin grafts with a 5mm biopsy punch, (B) shows the placing of the split skin grafts onto the CAM.

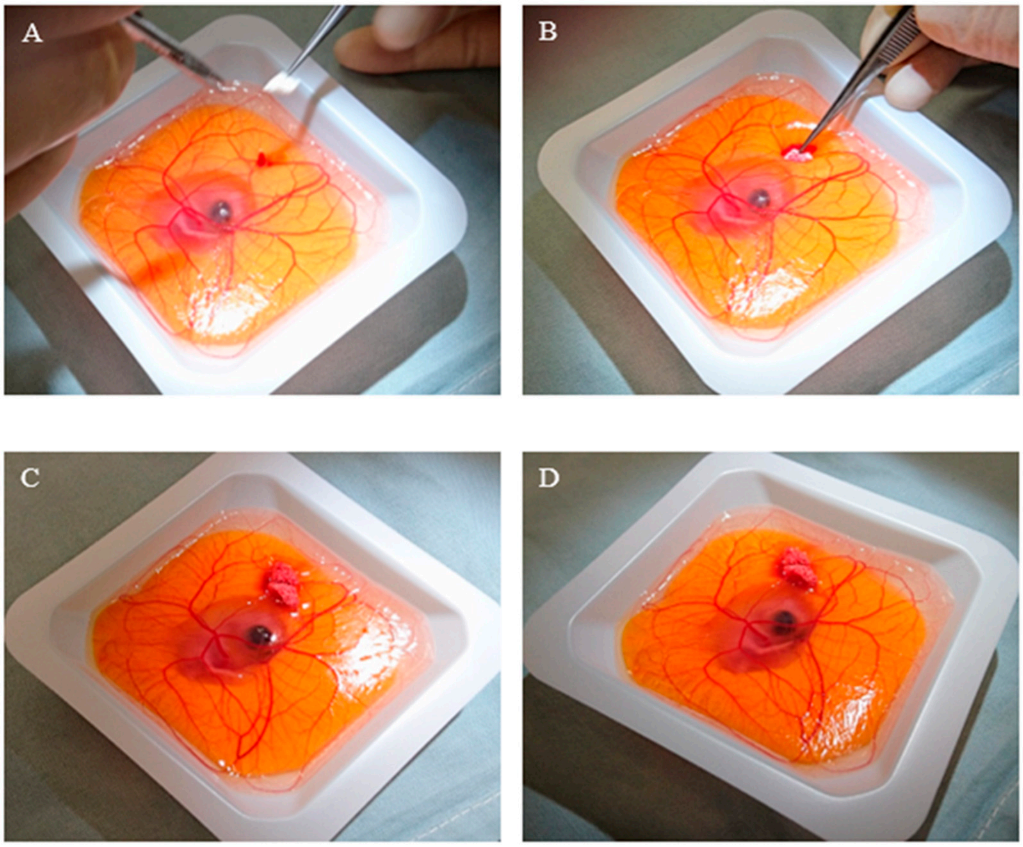

Figure S2. Blood-stopping method. Figure S2 (A) shows a bleeding CAM vessel on day 11 after injury, (B) how to press m-doc onto the bleeding vessel. In Figure (C),(D) the m-doc sponge is soaked up with blood and haemostasis occurs.
